# Lessons from COVID-19 outbreaks for spaces between buildings using tactical urbanism

**DOI:** 10.1186/s44147-023-00173-0

**Published:** 2023-01-13

**Authors:** Mai M. Abdelkader, Marwa Khalifa, Abeer Elshater

**Affiliations:** 1 Urban Design and Planning Department, Faculty of Engineering, Ain Shams University, Cairo, Egypt; 2 Faculty of Engineering, Ain Shams University, Cairo, Egypt

**Keywords:** Spaces between buildings, COVID-19 outbreak, Tactical urbanism, Comparative analysis

## Abstract

Several urban agendas related to different urban spaces in cities are documented in the global literature. This research explores social interactions in voids between buildings using tactical urbanism. As part of this study, we examine changes in perceptions of the use of spaces between buildings by comparing critical differentiation factors before and after the outbreak of COVID-19. We conducted an online survey for three months among residents in Egypt using a comparative method based on personal, residential, and district characteristics. The results revealed that during and after the COVID-19 pandemic, the spaces between buildings played a critical role. According to the conclusion, tactical urbanism, rapid and low-cost intervention, material availability, and small-scale pop-ups are essential for reducing the adverse effects of COVID-19. These findings confirmed that the longer the outbreak persisted, the more planning shifted to smaller public spaces within walking distance, resulting in long-term activities rather than large areas of land being planned.

## Introduction

The rapid spread of coronavirus pandemics worldwide has significant implications for the work-life balance and has far-reaching impacts on millions of people [8, 49]. COVID-19 is a global pandemic of unprecedented scope, scale, and influence, so it can be considered an assault on urbanity [14, 28, 45, 74]. Although COVID-19 is not our first pandemic, it is the first time the design has prioritised public health [36, 88]. Furthermore, it may provide an opportunity to incorporate health considerations into new ways of planning [50, 78].

The future of public spaces is ambiguous because, during the first three months of the outbreak, access to public spaces was restricted to reduce the rapid rate of outbreaks [66]. However, this process has proved to increase the desire for public spaces, but it is noteworthy that after the outbreak, the frequency of visiting public places has decreased significantly [67]. Moreover, there is a problem with the public space policy system as most of Cairo’s public spaces are privatised and fenced, and most of the territory is occupied by private cars [18]. It has caused considerable fear, worry, and concern in certain groups, such as the elderly, care providers, and people with underlying health conditions [47]. In addition to considering the current economic crises that would follow COVID-19, it is fair to say that place-making activities would not be on the top of the funding agenda since place-making can be a long and challenging process [5, 27]. According to this problem, the following questions are raised:

•What features of change in the usage of spaces between buildings before and after the COVID pandemic?

•What are the priorities of tactical urbanism principles that mitigate the negative effect of the current COVID-19 pandemic?

Recently, urban planners, designers, architects, and landscape architects have discussed how this crisis would transform our relationship with public space [4, 35, 71, 74, 79, 83]. Scholars from diverse disciplines discussed the effect of COVID-19 on different fields such as the global economy [21], information technologies and smart cities [3], education [57], public health and the food system [24], and environmental psychology [72]. However, there is little focus on the potential of spaces between buildings during COVID-19 in Cairo. Furthermore, more research is required to promote tactical urbanism principles in these void spaces. Unlike Western literature, if future waves of pandemic outbreaks are not as well defined as in Egypt, procedures and figures of readiness in global south cities should be considered.

COVID-19 has profoundly impacted public life, and people have attempted to return to their everyday lifestyles by taking precautionary and preventative measures [8]. As a result, decision-makers need to consider alternatives to adjacent spaces and those within walking distance of the buildings. The spaces between buildings can create vibrant, welcoming, and lively public gathering places instead of leaving them empty. Hence, resorting to tactical urbanism principles adds value beyond physical activity and social interaction. The role of tactical urbanism is to improve the vitality of shared spaces and strengthen social interactions. This is done by identifying to what extent the shared space can meet citizens’ daily needs, such as communication, shopping, walking, and entertainment [52]. Moreover, tactical urbanism strengthens the sense of space within a neighbourhood, which can serve as a guide in creating convivial shared environments and understanding the relationship between design and recognition of areas in urban surroundings [33, 44].

This article aims to present a comparative analysis of changes in shared space use between buildings before and after the COVID pandemic. It also revisits the priorities of tactical urbanism in Cairo. This paper focuses on the user’s changing perception and usage of spaces between buildings after the pandemic. Using tactical urbanism approaches, restrictions on the use of shared spaces between buildings and social distancing policies have been lifted. In light of the current global health crisis, this study raises a critical question to guide future research and policy. Given the preceding, it is necessary to conduct further studies in Egypt.

The findings of this study may assist urban planners, urbanists, and policymakers in understanding the challenges and issues that will need to be addressed in the future. A contribution here highlights the importance of using the spaces between buildings as an alternative to public areas during and after an outbreak. Furthermore, tactical urbanism was promoted to overcome the negative aspects and issues that prevented people from spending time in these spaces. To effectively address the needs of neighbourhoods where people's lives and livelihoods are under threat, professionals and advocates from economic development, public health, criminal justice reform, and public space design and management must work together. Furthermore, the people affected by the pandemic must be directly involved in the response. This would lead to the implementation of such a controversial concept in our Egyptian context. Hence, our suggested results would lead to a better quality of life for Egyptian citizens and visitors.

This study is structured into four sections, which follow this introduction. The first section displays a literature review to help think beyond the current measures. This section considers the changes caused by the pandemic and how states and cities have responded to the coronavirus pandemic. In the second section, a conceptual framework is then drawn from this literature review. An online survey conducted by users seeks to answer our research questions. The third section shows the results and discussions. Finally, conclusions and future work are discussed.

## Literature review

This section discusses two issues: the spaces between buildings and the COVID-19 pandemic’s impact on these shared spaces. This section also shows how tactical urbanism principles could help lessen the destructive effects of the pandemic and encourage people to talk to each other.

### Potentials of spaces between buildings during the COVID-19 pandemic

**Fig. 1:**
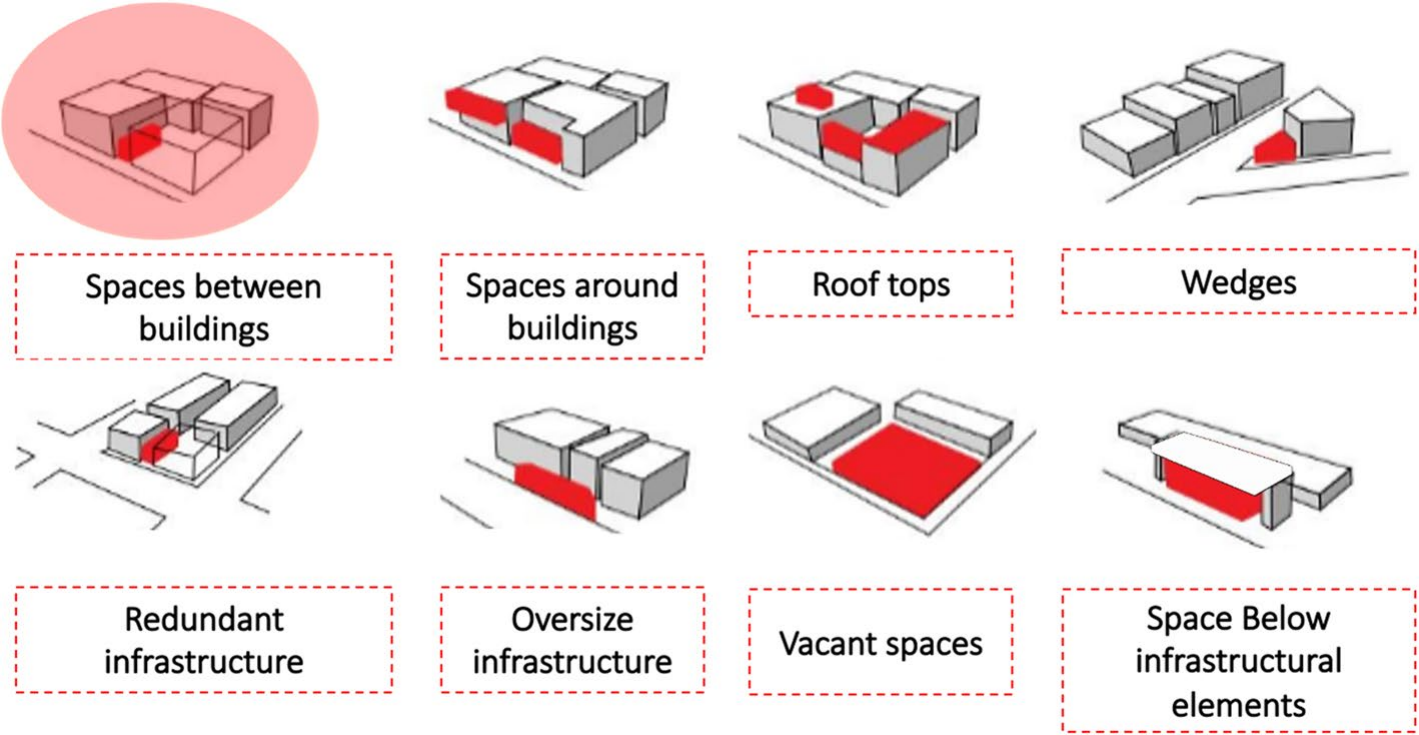
Classification of residual spaces and space between building as scope of work

This article first defined the spaces between buildings. The relationship between facilities and outdoor space has often been ignored and abandoned [22, 89]. One of the eight types of residual areas classified by [84] is the most abundant type of residual space that offers some of the most exciting opportunities for utilisation according to their different dimensions in width and length and other needs, as shown in Fig. 1.

The spaces between buildings are defined in this study as continuous spaces between different urban structures with various forms of mixed-use and development [59]. The spaces between buildings are urban passing moments that generate transitory shared spaces [16]. They are large or small-scale spaces, public or semi-public, in a state of transition—a pause—in functionality. They were described as the cracks in the city, residual, underutilised, and deteriorating spaces [56, 77]. However, spaces between buildings were mentioned as spaces that can be appropriated for creative uses but have no relation to ownership, size, type of use, or landscape character [1].

Cities have gone through more than one peak of the COVID-19 outbreak. The crisis’s size, scope, and speed make it feel like we live through a profound transformation [66]. Therefore, it is necessary to think more expansively about the everyday spaces that can fulfil people’s daily needs and work directly with the most affected communities by the virus and its economic consequences [8]. Hence, the management of outdoor spaces between buildings should be guided by updated evidence to limit the spread of the COVID-19 pandemic [19].

Lefebvre [51] classified everyday interventions as related and join functions, while Sheringham, in 2006, states that daily practice represents the work of the individual and social human being in society. Children and adults put their hands on to make a positive change during the interventions [9, 29, 75]. Users start to identify and analyse their environment according to necessities and desires. People usually use shared spaces by assigning them according to their activities and usages, consciously or spontaneously [10].

In congested urban environments, these underutilised spaces typically serve as breathing spaces. With its mix of retail, food, entertainment, and service purposes, these spaces provide an opportunity to engage in a variety of activities such as shop extensions, praying, seasonal activities, playing football, or going cycling. Social interactions were also recognised as meeting new people and making new friends. The outdoor seating area was used for conversation and gatherings. These activities allow users to achieve a reasonable degree of meaning and attachment to the space [23].

**Fig. 2:**
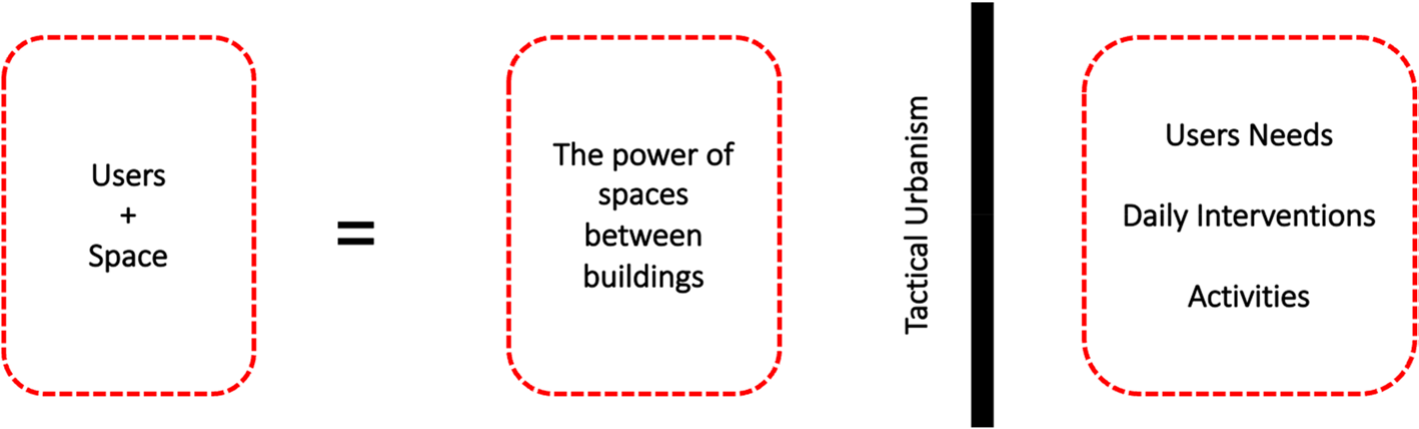
Revitalisation frameworks for spaces between buildings

Therefore, the users can convert a space into a particular living place. Tactical urbanism or do-it-yourself urbanism (DIY) refers to interventions carried out by the users of the area to enhance the surrounding environment. These interventions can occur by individuals or groups to fulfil their everyday needs [46] (see Fig. 2). Teresa [80] describes the design interventions as temporary, guerrilla, pop-up, DIY, small-scale projects, or tactical urbanism because all of them were willing to achieve a social impact. As a small action, a design intervention involves citizen participation in transforming underutilised spaces in the city according to citizens' interests, needs, or opportunities. Other researchers recommend that both citizens and local institutions should be invited to these spaces [2, 30]. Furthermore, every neighbourhood also has an abundance of talents and passions that could make our streets more beautiful and livelier, even from a distance [60, 63].

### Tactical urbanism and the COVID-19 pandemic

Lydon and Garcia [53] define ‘tactical urbanism’ as an approach to neighbourhood building and activation using short-term, low-cost, and scalable interventions and policies. In this context, Coyle [27] describes tactical urbanism as a minimal-expense intervention, broadly accessible and adaptable materials, items, and constructions to rapidly make new types of shared spaces of any size. It functions as a temporary solution and can considerably impact the design and utilisation of these urban spaces, whether in Cairo or elsewhere in Egypt. Practical tactical urbanism takes several forms in Cairo’s urban areas and streets, from local annual festivals to weekly market events [2, 30]. One more type of these tactical exercises is youth and club initiatives dealing with urban and socio-economic development, leisure activities, or cultural movements. Public health and urban change can be advanced through these tactics [41].

COVID-19 mitigation measures can benefit from tactical urbanism interventions with a long-term health vision, which can boost the vitality of residual spaces between buildings. These interventions benefit from physical activity and social interaction [68]. Using basic materials such as flowerpots, new planting trees, and coloured paint creates a bit more space for people to ride bicycles or walk, thereby making walkable streets safer and more accessible for people [61, 69]. Tactical urbanism interventions take less time to design, plan and approve, so they generally work faster and provide the perfect short-term stimulus [41]. The phenomenon of social distancing creates humour and joy by proposing new activities that tend to create cheerfulness, things that users do not usually do in public [6, 8, 13]. Due to the movement restrictions of the COVID-19 pandemic, the relationship between citizens and their space has changed [81]. Public spaces need to be part of the virus response to provide people with the opportunity to relax and work [11]. The use of shared spaces between buildings can be transformed into pop-up community health centres or food gardens [24], as well as for organised street vending on specified days or times of the day or for leisure activities such as displaying films or plays or hosting exercise classes [43].

On the one hand, neighbours have come up inventive ways to stay in touch and remain associated, such as communicating across balconies or driveways, to prevent isolation [76]. On the other hand, these ways are crucial in decreasing stress, boosting well-being, and promoting the development of children [28]. By extending alleyways or enlarging bicycle lanes, the pandemic may develop new patterns and configurations of use [20].

Hence, a new vocabulary or typology by changing the type and distribution of green areas in spaces between buildings is a high demand that planners and designers should follow to create spaces refuge away from the hustle and bustle of the city where people feel welcome, comfortable, and safe [12]. As a result of the visual image of the city getting in touch with nature, residents appear to be benefiting physically and mentally. They are able to get their daily dose of nature in no time [19]. The long-term impact of COVID-19 provides an invaluable learning opportunity for developing tactical urbanism theory to learn where and why some of the changes have been implemented [50]. Hence, stakeholders will take health arguments in planning more seriously in a post-COVID-19 pandemic [66].

**Fig. 3:**
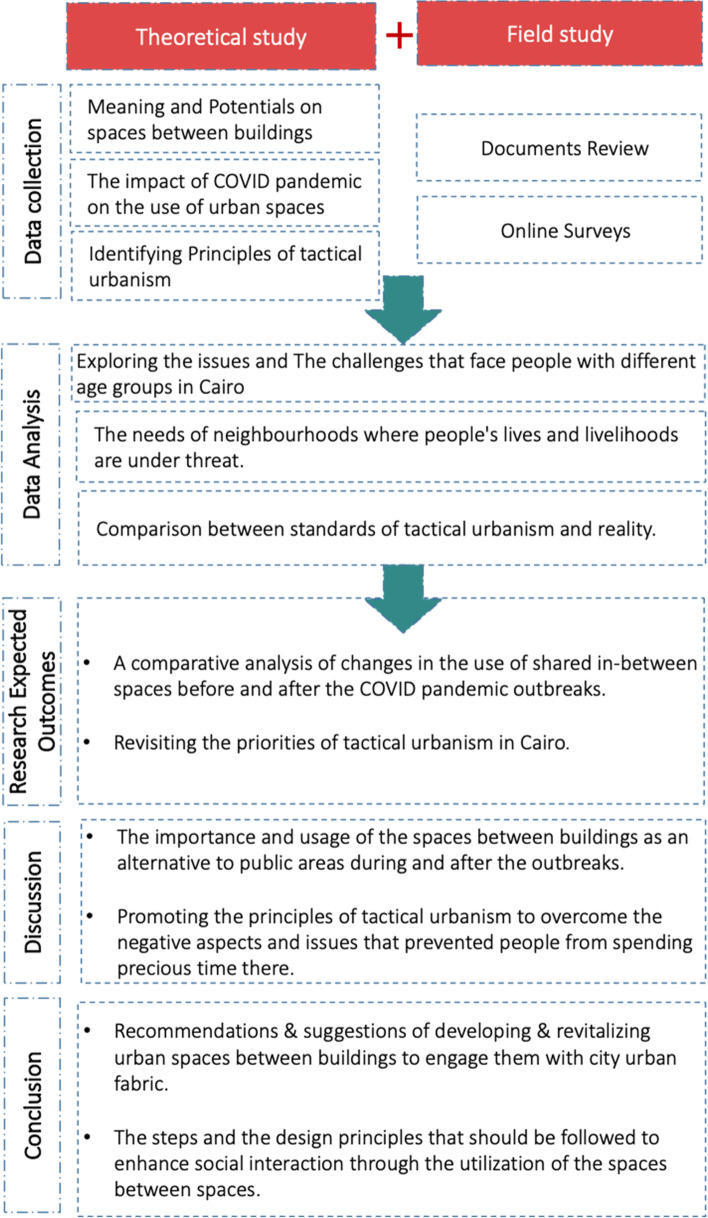
Research methodology and the analysis of spaces between buildings revitalisation process

After reviewing published literature concerning the research topic, the next step is to explore how to revive the residual spaces between buildings during the COVID pandemic according to previously mentioned principles, as shown in Fig. 3.

## Methods

This paper attempts to interpret the term ‘spaces between buildings’ and how such places promote social interaction. The research investigated the changes in perception and the usage of spaces between buildings that residents most frequently encountered before and after the COVID-19 outbreak in Cairo. The conceptual framework follows a quantitative approach to data collection guided by the leading research questions. This research was designed based on an online survey and collection of previously published research and online archival resources based on the work of different scholars. Within this scope, a statistical analysis was used with both primary and secondary data. As preliminary data, a survey was conducted with 277 participants living within the different areas in Cairo from March to June 2021. This research used a descriptive exploratory approach by choosing a random sample collected and considering the conclusion reached from reviewing the literature.

The survey questions were designed to be understandable by both experts and users. They include both closed-ended and open-ended questions. Closed-end questionnaires were standardised, quantifiable, and used to generate empirical data. However, due to the limited scope and time of research, the survey questions cannot be manually ported. Survey questions were designed using a user interface that leverages Google Forms. These datasets were quantitatively analysed using the IBM Statistics Package for Social Science (SPSS) to interpret differences between participant responses related to individual, place of residence, and district characteristics. To define the size of the sample, it was estimated using the following equation:

$$\textrm{Necessary}\ \textrm{Sample}\ \textrm{Size}={\left(\textrm{Z}-\textrm{score}\right)}^2\times \textrm{StdDev}\times \left(1-\textrm{StdDev}\right)/{\left(\textrm{margin}\ \textrm{of}\ \textrm{error}\right)}^2$$

where *Z* is the confidence level, assuming you have selected a 90% confidence level and equals 1.645. The acceptable margin of error that corresponds to the needed confidence level is equal to 0.05. The standard deviation is equal to 0.5. This equation is for an unknown or huge population size [79].

$$\Big((1.645)2\times 0.5\times \left(1-0.5\right)/{(0.05)}^2$$

Moreover, when using a broader context, saturation refers to the point in data gathering when no more problems or insights are discovered, data begins to duplicate, and additional data collection becomes redundant. The data collection reaches the appropriate sample size [26, 42]. Accordingly, over 200 were determined as an adequate sample size. The questionnaire was divided into two parts: the personal characteristics of the respondent section and the analytical section. The first part included gender, age group, education qualification, demographics, and occupation status. The second part consisted of eight questions that were classified into four types as follows:

- General perception and evaluations (regardless of the outbreak),
- Usage of shared spaces between buildings before the outbreak (past tenses),
- Use of shared spaces between buildings during the outbreak (present tenses), and
- Usage of shared spaces between buildings after the outbreak (future tenses).

The online questions about how the COVID-19 pandemic changed the design, use, behaviours, and perceptions in shared spaces were sent to the users on different social networking sites. This online survey was conducted in English and Arabic for better communication. The eight questions were:

1. How much time did you spend with your friends or family in the spaces between buildings one day before the pandemic?
2. In one day, when do you spend the most time with your friends or family in the spaces between buildings?
3. How satisfied are you with the design of the spaces between buildings in your neighbourhood?
4. Are these shared spaces safe and designed to be suitable for all kinds of users like older adults, children, and women to socialise and spend their daily needs in the morning or evening?
5. Can you recall an incident in the shared 'spaces between' that bothered you and made you dislike spending time there? Or is there a reason why you have never walked through these spaces?
6. Our perceptions of shared spaces changed after the current pandemic, so what features/kinds of change?
7. What features do you/your family need or enjoy most spaces?
8. Are you with or against planners beginning to prioritise the design of smaller local neighbourhood green spaces over large open spaces?

All the respondents’ identities remain confidential, and they were presented with consent forms before participating in the study. Thus, this section provides an inductive process from data collection to analysis, leading to the design intervention using tactical urbanism tools and focusing on using daily tools to analyse the open shared space. These tools helped uncover how space is conceptualised and used by different actors providing the main inputs for better using and utilising the spaces between buildings and what opportunities lay behind these spaces.

## Results and discussion

Two indicators were used to identify the usability of spaces between buildings on one hand and users and activities on the other. The indicators of user aspects were the personal characteristics of respondents. The activity indicator aspects were the range and intensity of the usage of spaces between buildings and the user’s associated evaluations. The pilot study of 277 participants consisted of 135 working people. The participants comprised 132 females and 145 males of different ages and education levels. The results indicated that all age groups were doing their activities in these spaces according to their perception, from youth to the elderly. The other important finding of the survey was that there was a significant difference between age groups regarding the frequency of going to the spaces between buildings. As such, the main descriptive statistics derived from the questionnaire are summarised in count and percent in Table 1.

**Table 1:** Personal and residential characteristics of the survey respondents

	Personal characteristics	Frequency	Percentage
Gender	Female	132	47.7%
	Male	145	52.3%
Age	20–25	87	31.4%
	25–35	86	31.0%
	35–45	56	20.3%
	45–60	30	10.8%
	Above 60	18	6.5%
Educational qualification	University student	87	31.4%
	Bachelor’s degree	119	43.0%
	Master’s degree	44	15.9%
	PHD	27	9.70%
Residence	Cairo	76	27.4%
	Giza	59	21.3%
	New Cairo	54	19.5%
	New urban communities	70	25.3%
	Outside Cairo	18	6.50%
Working status	Working	135	48.7%
	Not working student	75	27.0%
	Not working—housewife	22	7.9%
	Looking for a job	35	12.6%
	Business owner	10	3.8%

According to the results, many developed urban communities have niches and ‘spaces between buildings’ that support stationary activities. The results showed that activities were primarily found in the inner parts of the modern settlements. However, static activities such as sitting and standing tend to occur mainly in the spaces between buildings. This result was aligned with Gehl [38], who mentions that 70% of long-duration activities happen along the soft edges of spaces between buildings. Before the outbreak, most users, particularly men, spent 1–4 h in these spaces, but a higher percentage of female users spent less than 1 h, as shown in Table 2. The peak time for women’s activity was in the morning, while men were in the evening. Unlike Meier [58], the facts indicate that the spaces between buildings had high usability in a shorter time, not reaching 24 h of usage a day, as shown in Table 3. This shows the importance of paying more attention to the residual urban spaces to spend more time in daily life as a catalyst for restoration, which ultimately affects people’s life and communication.

**Table 2:** The frequency of going to the spaces between buildings before the outbreak of gender differences

Levels of abstraction	Gender	Frequency of spending time in the spaces between buildings before the outbreak	Percentage
Between 5 and 10 h	Female	17	6.10%
	Male	28	10.1%
Between 1 and 4 h	Female	38	13.7%
	Male	56	20.2%
Less than one hour	Female	50	18.1%
	male	37	13.4%
Never go there	Female	31	11.2%
	Male	20	7.20%

**Table 3:** The time you spend more in the spaces between buildings in one day

Levels of abstraction	Gender	Frequency	Percentage
Morning	Female	61	22.0%
	Male	49	17.7%
Evening	Female	45	16.2%
	Male	77	27.8%
Never go	Female	28	10.1%
	Male	17	6.2%

Based on the results of the previous question, we found that we need to measure the level of satisfaction with the design of the spaces between buildings in their neighbourhood. Our results were yielded by using a five-point Likert scale ranging from 1 indicating very dissatisfied, 2 indicating dissatisfied, 3 indicating neutral, 4 indicating satisfied, and 5 indicating very satisfied. Using the scale’s mean value equals 3 is the midpoint. Therefore, values above 3 are considered somewhat comfortable but at higher levels. Similarly, any value less than three is unsatisfactory but at a lower level.

**Fig. 4:**
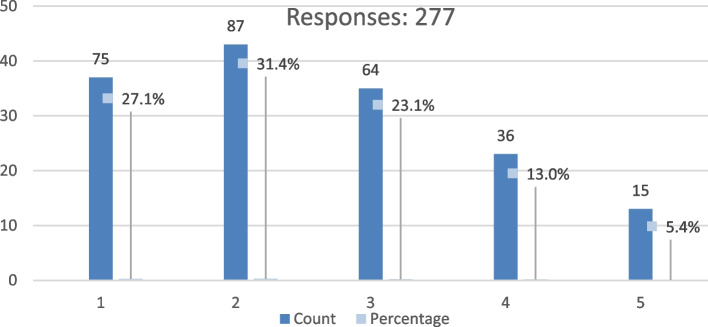
The measure of satisfaction with the design of the spaces between buildings in his/her neighbourhood

However, the results showed that the respondents who asserted ‘unsatisfied’ (scores 1 and 2) were more significant than those who claimed ‘satisfied’ (scores 4 or 5), which means they were not satisfied with their spaces between buildings in Fig. 4.

A community sense of attachment is crucial because it suggests that how change takes place in communities may also have the potential to produce social benefits [13, 37, 54, 55]. This research is more concerned with quality-of-life factors about people’s activities in the shared spaces between buildings. The quality-of-life factors analysed health, recreation, and urban environment [27, 73]. The results supported the levels of these spaces that should be planned to meet the residents’ satisfaction and sense of attachment [58]. As such, satisfaction with open shared spaces relates to satisfaction with the overall quality of life [6, 34].

**Fig. 5:**
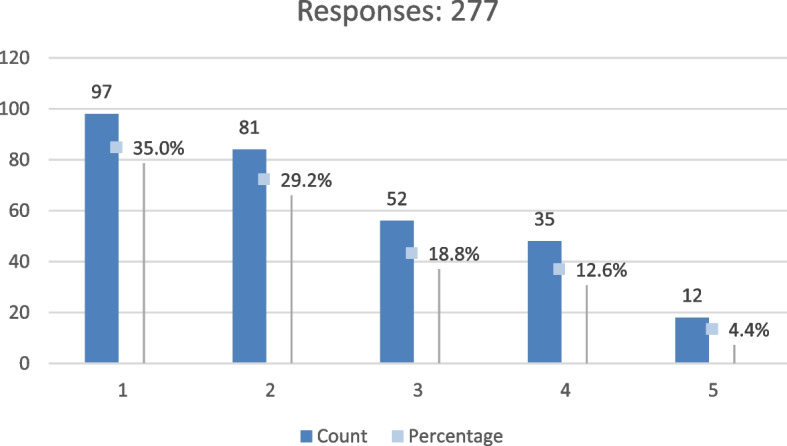
The level of safety with the design of the spaces between buildings in their neighbourhood

This result is in line with previous research where Minou, Dane, and Berg [86] stated in their questionnaire that overall, people are satisfied with urban public spaces—considering that their discussion was about public spaces in the City of Eindhoven, not the shared spaces between buildings. To ensure the results of satisfaction levels, the questionnaire also discusses the level of safety and if the spaces between buildings were designed to be suitable for all kinds of users like old people, children, and women to spend their daily needs in the morning or evening also used a five-point of Likert scale, the results showed that the majority who asserted’ significantly disagree with the safety of these spaces (score 1) were more significant than those who claimed’ (scores 4 or 5) which mean that the majority of spaces between buildings are ineligible for the user’s needs as shown in Fig. 5.

The results also were confirmed by Honey-Rosés et al. [43], who discussed the level of safety. They stated the absence of safety and security, as criminal groups have taken functional control of public spaces and women’s access to shared space has been impacted by the consequences of the outbreak. These findings concur with those identified by the National Vacant Properties Campaign claims that vacant residuals between buildings endanger public safety and frequently show signs of property owner neglect [58, 87]. In addition, previous research mentioned that safety and adequate places, such as kids’ areas for children to play are important as the appropriate environmental characteristics that are suitable for outdoor play [25, 32, 40, 82].

The spaces between buildings have a positive potential, particularly if they are suitably enacted. However, numerous spaces between buildings represent a danger to safety and security if they may no longer be utilised intentionally [15, 62].

As a result, the survey should clarify the everyday incidents that may occur in these spaces that cause users to become irritated and dislike spending time there. We used checkboxes to display the answers to this question to analyse the main issues with the spaces between buildings that face different age groups.

**Fig. 6:**
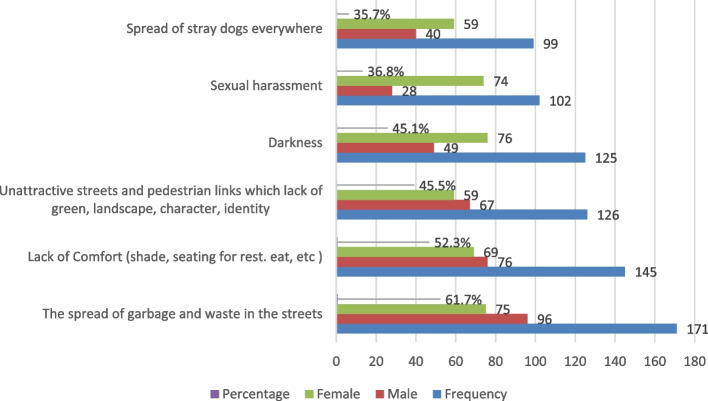
The fears and the incidents that faced users of the spaces between buildings

This study showed that most male or female respondents were 61.7% annoyed by the spread of garbage and waste in the street, which is considered one of the main issues that users face. These results are compatible with the studies conducted by Azhar and Gjerde [15, 16] which asserted that these unused small spaces between adjoining buildings often attract trash and waste. Approximately 52.3% of the results are dissatisfied with the lack of comfort (shade, seats for rest, eating). About 45.5% suffer from unattractive streets with a lack of green areas and the elements of landscape architecture, as shown in Fig. 6. However, the feeling of comfort is one of the critical aspects that illuminates the shared spaces. It is seen as a positive emotional reaction to external surroundings in different situations, including physical, social, and psychological responses, and the degree of feeling comfortable is dependent on the surroundings, which are closely related to the duration of stay and the frequency of use [85].

Nearby, the other responses suffer from safety, classified as fear of darkness, sexual harassment, and the spread of stray dogs everywhere, which is compatible with the incidents discussed [39]. Losing the sense of place and feeling unsafe and anxious clearly impact residents if the spaces between buildings in the neighbourhood remain dead [70]. The results support what Jacobs and all subsequent scholars have suggested about the importance of shared spaces between buildings in promoting a sense of safety and security through more eyes on the streets [6, 17, 48].

In terms of the problems and incidents faced by residents and users of the spaces between buildings, the outbreak of the COVID-19 epidemic is sweeping the world and has positive and negative impacts on the use of these spaces. Therefore, the next part of the research analysis is the COVID-19 pandemic’s effect on the frequency of going to the spaces between buildings. According to the responses, the researchers of the present work found that 19.5% of men spend the same time with taking into consideration some precautions, and about 15.2% of men spend more time than before the outbreak, unlike 17.7% of women spent less than the time before and 11.2% no longer spending time there. This indicates that the effect of the trauma caused by the pandemic would continue for a long time. However, the outbreak of COVID-19 appears to be a turning point that will permanently change the perception. How personal, residential, and regional characteristics affect the perception and use of these shared spaces. Above this level of satisfaction, people kept doing their activities in these shared spaces and making them an active area.

In terms of residential and personal characteristics, particularly gender [1], there is a significant difference in the use of spaces between buildings before and after the outbreak. There is a decrease in the frequency of women using shared spaces between buildings after the outbreak compared to men, who spend a higher percentage of their time there after the outbreak, as shown in Tables 2 and 4. The results demonstrate that the pandemic has more negative impacts, especially on women who use shared spaces, as confirmed by findings raised by Paköz et al. [67], who focused on public spaces rather than shared spaces between buildings. Before the outbreak of COVID-19, it was shown that the possibility of women's visits to public places was reduced. They spend more time there after the outbreak than men.

**Table 4:** The change in the frequency of going to spaces between buildings during the outbreak of gender differences

Levels of abstraction	Gender	Frequency of spending time in the spaces between buildings after the outbreak	Total Percentage
Less than the time spent before	Female	49	17.7%
	Male	30	10.8%
Spending the same time with keeping their distance from each other	Female	29	10.5%
	Male	54	19.5%
No longer spending time there	Female	31	11.2%
	Male	19	6.80%
Others (-Spend more time)	Female	23	8.30%
	Male	42	15.2%

**Fig. 7:**
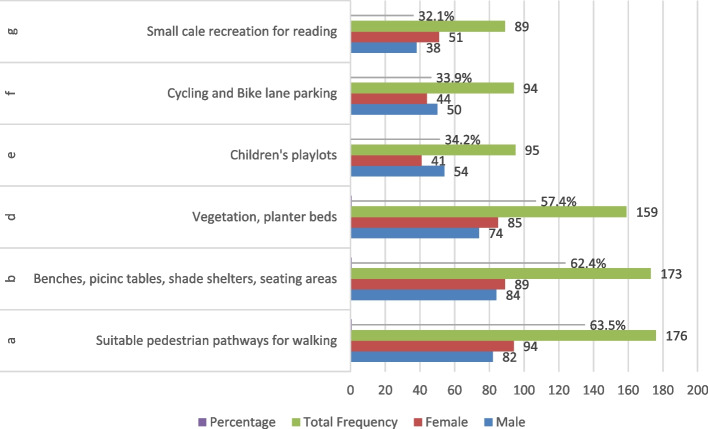
Priorities of the principles of tactical urbanism in future interventions

The community engaged in both active and passive activities, such as walking, talking, and conversing with friends and family members, eating and drinking, and entertainment (picnicking, playing, cycling, and children’s play areas), or simply sitting while enjoying the open greenery space features and environment [64]. There is no significant difference between males and females in ranking the six activities they need in the spaces between buildings, as shown in Fig. 7. To be more focused, it is essential to list the user’s preferred activities and arrange the principles of tactical urbanism to focus on the user’s priorities and preferences while starting future initiatives and interventions to increase the vitality of shared spaces between buildings.

These activities promote a sense of place and have the most significant potential to empower the community to drive future change. People can also create a place they will be proud to be associated with in the future [31]. Thus, the residents would meet frequently and feel more stable in the neighbourhood. Social interaction will occur with many eyes on the streets to improve safety [74]. These results also affirmed Newman’s intention to create a defensive space guideline to allow the community to manage spaces previously thought to be inaccessible public spaces. Residents care about their space as much as they care about protecting their private property and themselves from crime [65]. However, other critical issues, such as connectivity to other areas and the surrounding city fabric, should be carefully considered [36].

**Fig. 8:**
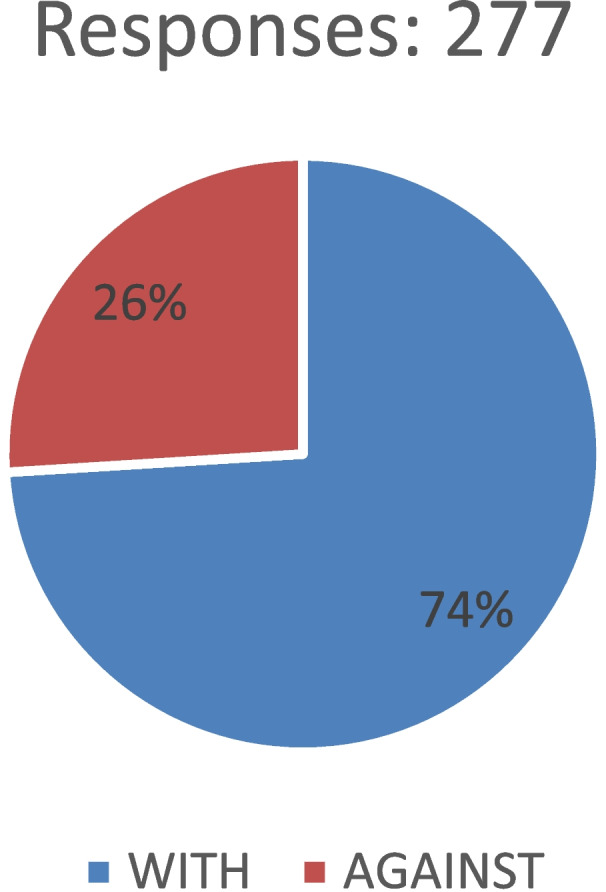
The priority is to design smaller local spaces between buildings rather than large open spaces

Our survey results reveal a significant shift towards using common spaces during the outbreak. The responses also show that a significant proportion of the participants believe this shift should be permanent in the post-pandemic city. According to all the above questions, the last one sums up if the user is with or against urban planners beginning to prioritise the design of smaller local spaces between buildings and neighbourhood green spaces over large open areas. The results showed that the majority of people (74.0%) agreed with the concept of prioritising the design of local spaces between buildings over larger open spaces, as shown in Fig. 8, for several reasons. The first is that, during a pandemic, the spaces between buildings are now proven necessary because most public areas may be closed again due to security procedures. People need a clean environment with aesthetic views and increased green space per capita, ensuring an equal share of green space for everyone. The prominent places will come in a second phase.

**Fig. 9:**
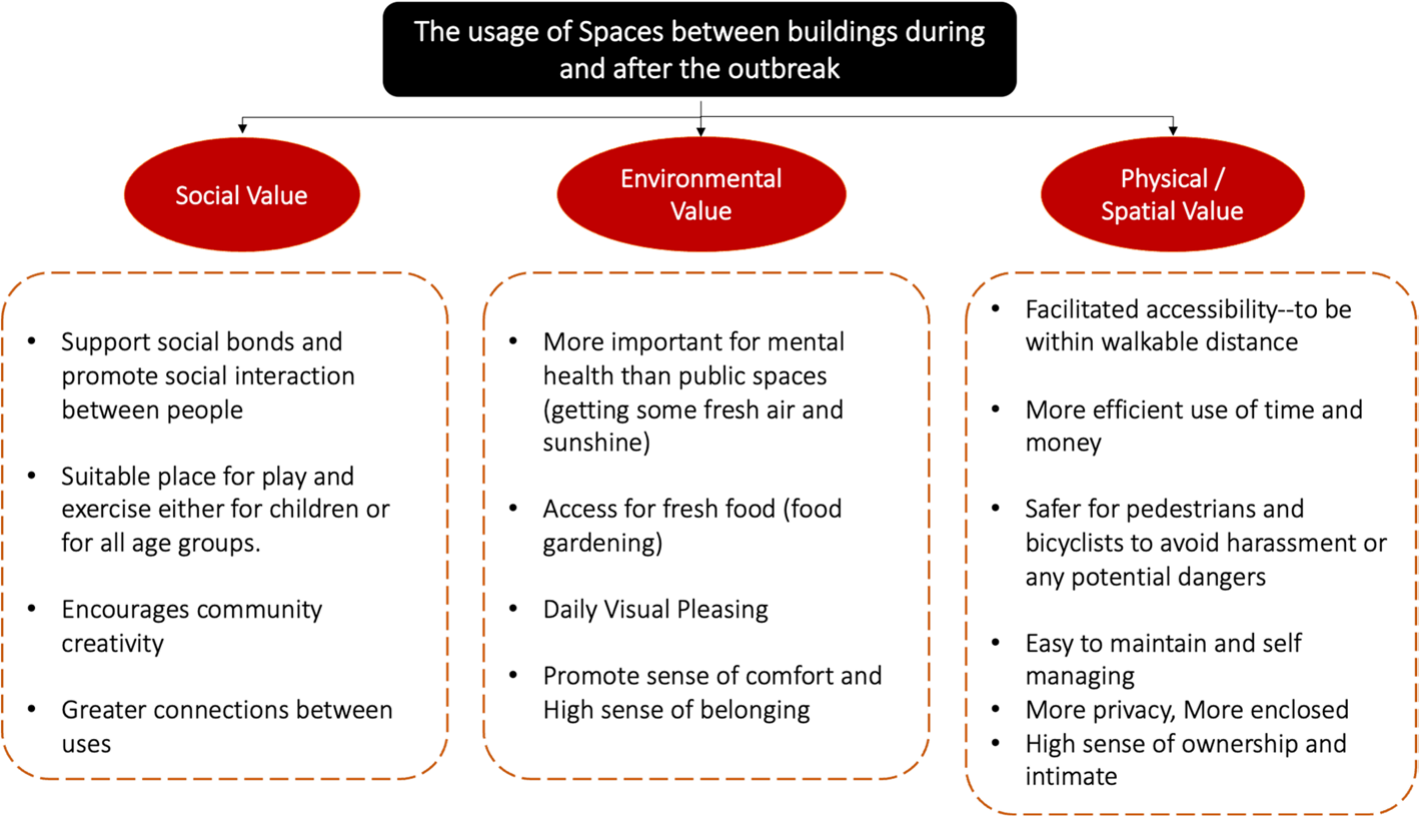
The usage of spaces between buildings

The second reason was facilitated accessibility by being within walkable distance, which was confirmed by the high percentage of walking activity for daily activities. The third reason was giving opportunities to the local community, supporting social bonds, and promoting social interaction between people. The fourth one was for more safety to avoid harassment or potential dangers. The fifth one was a high sense of ownership, more privacy, more enclosed intimacy, and a heightened sense of belonging. It is also easy to maintain and take care of, which leads to the preservation of the surrounding environment. Summarising all the above, the spaces between buildings will improve the quality of their neighbourhood, which will positively affect their psychology and mental health. It has been demonstrated that citizens can survive and adapt to crises with appropriate spatial policies. Suggestions and the fulfilment of recreation needs are city-of-life indicators [41], as seen in Fig. 9.

**Fig. 10:**
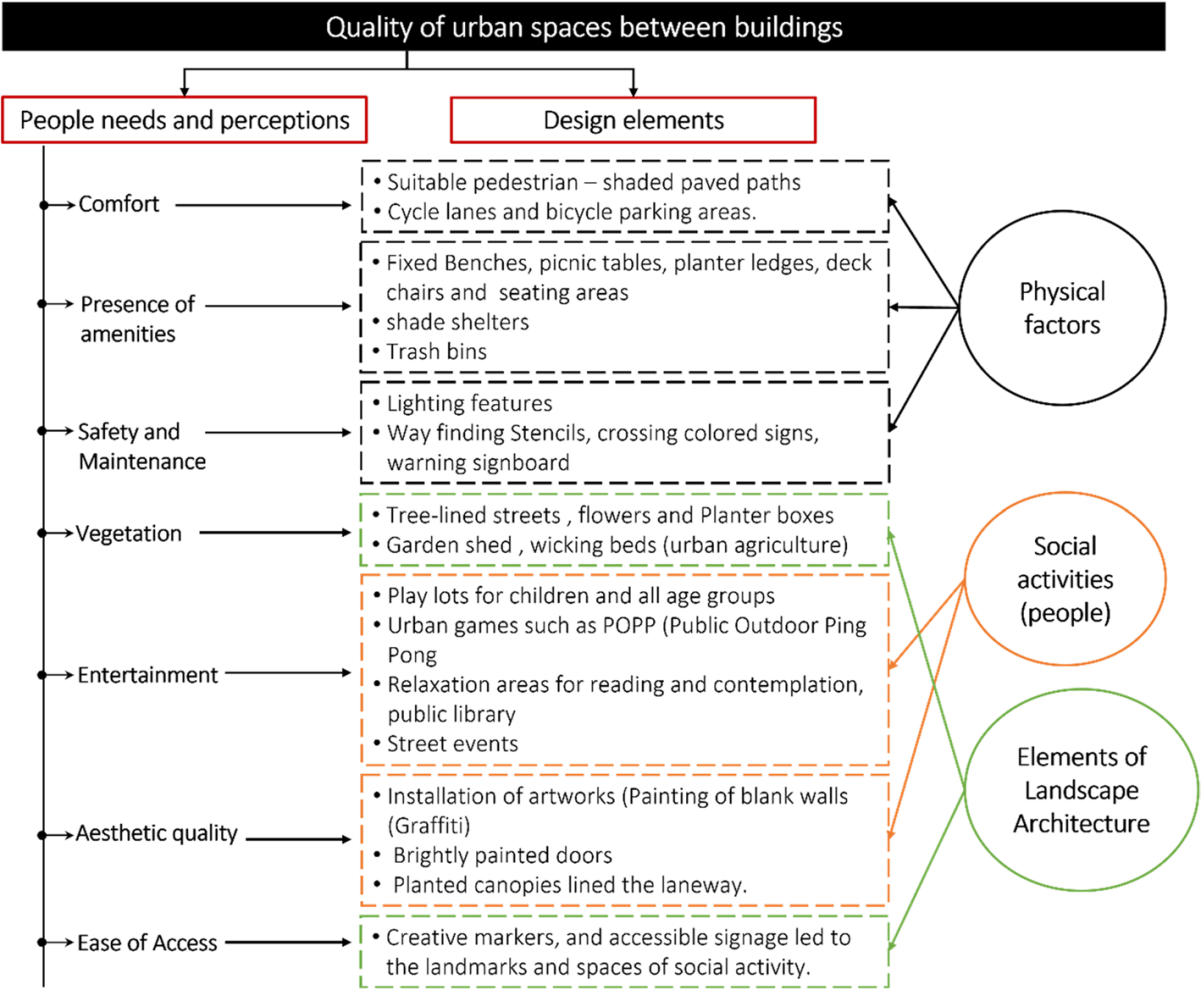
Design intervention

This research recommends ‘Do It Yourself’ interventions according to users’ priorities and results to be permanently implemented to benefit people more broadly in the long term and combat the COVID-19 pandemic, as shown in Fig. 10 .

Due to time and resource constraints, the research only studied a subset of residents in random residence areas affected by the current COVID-19 pandemic. Therefore, specific areas should be considered and targeted based on understanding the context to enhance and strengthen the results. Collecting data from more participants would have provided a complete understanding of the subject. Due to a lack of ability, a larger sample size should be considered, and different neighbourhoods within the city should be studied to generalise and validate the overall Cairo findings. Furthermore, the analysis should have addressed governmental urban plans and policies because it focuses solely on residents' experiences and perceptions. The research participants included only residents and no governmental representatives or other stakeholders.

Based on the current research context, the survey was conducted between March and June 2021, just after the first phase of the pandemic. Therefore, the results should reflect the perception at that time. Repeated investigations in other states may result in different outcomes. Egypt’s warm climate allows the use of open spaces throughout the year. Since many human activities depend on weather conditions, similar studies are needed to cover different phases of the outbreak in Egypt and different cases worldwide. Further studies that can contribute to understanding the shaping and transformation of public space in the post-pandemic city are expected to deepen these inquiries and discuss them in different periods and cases. Therefore, this study points out that pandemics can cause changes and revolutionise the perception and use of shared spaces between buildings.

One of the significant limitations of the study was that the time available to conduct the chosen methods needed to be increased to achieve optimal results. Additionally, online questionnaires were answered only by a minimal number of people who are familiar with using the internet. Hence, using online questionnaires does not help reach a broad sample of people. Due to the limitations mentioned in this paragraph, the results were relatively poor. Furthermore, because the questionnaire is a quantitative method, it cannot be covered without qualitative sociological tools such as observation. At this level, you can extend the snapshot tool application to a natural environment to understand the social use of your neighbourhood in its urban entity.

## Conclusions

This paper investigates possible changes in perception by comparing differences in the use of shared spaces between buildings in Cairo before and during the pandemic outbreak. In addition, the paper highlighted the importance of designing spaces between buildings as a new medium for social interaction instead of large public spaces. This research assessed the tactical urbanism approach using its principles. This study started with a general overview of the spaces’ characteristics and components, to a detailed exploration and analysis of the everyday spaces before and after the outbreak. Mental mapping of the old and new residents’ perceptions of their shared spaces was also essential in relating to their imagination and affiliating with the neighbourhood. An online questionnaire was designed to investigate the potential of spaces between buildings in the community and comprehend the effects of COVID-19 on these informal social interactions.

This study also concludes different levels regarding utilising the shared spaces between buildings and thinking beyond the current measures to consider the priorities of tactical urbanism principles during and after the pandemic outbreak. The results reveal that as the pandemic gets longer, the possibility of giving attention to spaces between buildings to change urban habits increases. The online survey, conducted with 277 participants, lasted from March to June 2021. The present study presents the differentiation of this effect according to personal, residential, and district features. This condition can continue to a large extent after the outbreak. Hence, tactical urbanism adds value beyond physical activity and social interaction as it increases streets’ vitality and enhances a local sense of place close to residential areas.

This study contributes to the debates about the future of shared spaces and opens new discussions on how shared common areas can be transformed and designed in post-pandemic cities. The most important recommendation for future research is to achieve more accurate and robust results. They should build a better foundation to minimise the limitations that face this research. We also anticipate that circumstances will change between the time of writing and the time of reading. Things are moving fast. As a result, an early start should be considered to allow as much time as possible for methods to be implemented and results to be met. The other vital recommendation is to use more techniques than online questionnaires to reach a broader sample of people and get a more extensive range of feedback and opinions.

As per our findings and discussion, the space plan needs to function to improve the common shared spaces between buildings to be suitable for daily or weekly uses. The current study concentrated on everyday activities. Other factors can be explored and may affect the use of spaces between buildings. The duration of residence, homogeneity or social and functional heterogeneity can be among the aspects that can influence the use of outdoor spaces between buildings.

Building on the research limitations, it is likely that other theoretical frameworks will be tested against tactical urbanism, given the abundance of spaces between buildings in many cities, to realise the full potential of underutilised urban areas and contribute to the well-being of its residents. A multidisciplinary, behavioural, sociological, and environmental approach can be considered to explore the other aspects over the medium or long term to identify the impact of time on the usage of spaces between buildings.

## Data Availability

The datasets used and analysed during the current research are available from the authors upon reasonable request. The authors confirm that the data supporting the findings of this study are available within the article and its supplementary materials. In addition, the data of the questionnaire analysis was done with the assistance of Google Forms and are available on Google Drive through the link: https://docs.google.com/forms/d/e/1FAIpQLSeMyIqKkDacErQTalgQS3Bnfr6M0FHea8GYHqA98_nbuXT8hg/viewform?usp=sf_link

## Abbreviations

COVID-19: Coronavirus disease 2019
SPSS: Statistical Package for Social Science
DIY Urbanism: Do It Yourself Urbanism
